# Artificial Intelligence in Medical Science: Perspective from a Medical Student

**DOI:** 10.31729/jnma.5257

**Published:** 2020-09-30

**Authors:** Gaurab Mainali

**Affiliations:** 1 Nepalese Army Institute of Health Sciences, Sanobharyang, Kathmandu, Nepal

## INTRODUCTION

Artificial Intelligence has been an essential tool in our daily life. From playing YouTube video through Alexa or Siri to screening nodules for malignancies in medical science, it has shown its great utility in today's world. Artificial Intelligence (AI) is the ability of a machine to independently replicate intellectual processes typical of humans.^1^ To perform such function, it has many components integrated with each other. In medical science, one can focus mainly on image processing, computer vision, machine learning, artificial neural network, convoluted neural network, and deep learning.

Machine learning is the ability of a computer to learn from the previously recorded data through Artificial Neural Network (ANN). Inspired by the neural network of the human brain, ANN is non-linear relation between Input and output through algorithms between them. Deep learning is a subset of machine learning which is structured similar to human brain processing, taking into account multiple data sets at the same time, which are evaluated and reprocessed for second and third different evaluations and so on, until reaching an output.^2^ They can learn from past examples, analyze non-linear data, handle imprecise information, and generalize enabling application of the model to independent data.^3^ In deep learning Convoluted Neural Network (CNN) is used. CNN is a branch of Artificial neural network which contains many hidden layers of algorithm interlinked to each other to give the result. This workflow continues with multiple layers as needed (hence the term deep learning) while each filter creates an output score which is the input score of the next layer until a final result is achieved.^2^

## AI IN MEDICINE

In medicine, a well knowledgeable and experienced physician can make a proper diagnosis, instill proper treatment, and provide quality patient care. Knowledge of the proper evidence-based decision gives rise to good evidence-based medicine, the best way to practice medicine. Evidence-Based Medicine represents the integration of clinical expertise, patient's values, and best available evidence in process of decision making related to patient's health care.^4^ To give a more accurate and precise conclusion, the physician should review previously done diagnosis, treatment, and records. But there are many data and records that make it impossible for a physician to interpret and give necessary outcome in a given time frame. So, having the ability to harness these enormous data and records and transform it into an experience, AI is growing and taking an important place in medical sciences. Artificial intelligence (AI), and in particular deep learning, is among the leading technological tools beginning to be used in the interpretation of medical images and electronic health records.^5^ It can analyze millions of data in a short period for which physicians would have taken many years.

## HISTORY AND PROGRESSION

Using computers to imitate intelligent behavior and critical thinking, was first described by Alan Turing in 1950 with a test called Turing test. Later in 1956, John McCarthy coined the term Artificial intelligence (AI).^2^ In medical science, few AI related advancement had been done in early days of its evolvement due to limitation in AI. In early 2000, with the advancement of Technology in AI and the invention of Machine learning and Deep learning, Medical science welcomed AI and integrated it as a part of it for advancement in the medical field. Having proper diagnostic accuracy, improved efficiency and procedure accuracy, and better overall patient outcomes, AI is widely accepted by medical science. In 2016, AI projects related to healthcare attracted more investment than any other AI related sector of the global economy.^6^

AI is trained with data of previously made decisions, treatments, patient outcomes, drug development, prescreened image, identified features, radiographic characters which will be a baseline for identification and interpretation of new input data.

- In Oncology: AI is being used for diagnosis and grading of cervical carcinoma, breast cancer, and glioma; IBM Watson oncology for cancer care and treatment; CURATE. AI for automatically modify drug dose with the progression of disease in a cancer patient.^7^
- In Gastroenterology: Computer Aided Detection (CADe) is used in Endoscopy and Colonoscopy to detect lesion in the Gastrointestinal tract whereas Computer Aided diagnosis (CADx) interprets the image and provides a final interpretation of lesion. So, it is useful for screening the polyp, gastric carcinoma, gastritis. Wireless capsule endoscopy with deep learning detects bowel bleeding, hookworm, and other infectious diseases of GI tract.^8^
- In surgery: It is used for screening the surgical patient with the non-surgical patient. Preoperative analysis of patient data provides the risk score for the operation and prevents the complication.^9^ Though computer science entered in operating room through robotic assisted surgery, it is not associated with Artificial intelligence.^2^

The working efficiency of these AI is similar or more than that of human efficiency. Thus, it can be used in the medical field with some limitation.

## LIMITATIONS AND CHALLENGES

AI analyzes and gives results based on data that is provided to it. If the data and type of data are wrong or misinterpreted, it gives rise in a blunder in the diagnosis and treatment procedure. Moreover, most of the trained data in AI are already diagnosed, treated, and are from the past medical experiences so new cases and features cannot be read and interpreted by the AI. Ethical issue in medical practices is another limitation of AI. The main challenge for AI is that people are using a search engine as a virtual doctor for advice and consultation for their disease so, it can create misunderstanding with the human physician.

## WAY FORWARD

Artificial Intelligence, and particularly deep learning, increase the efficiency of treatment, increase doctor-patient interaction by reducing paperwork and increase safety in health care. With modifying limitation and proper monitoring of AI, it is growing in other applicable fields of medicine. The decision given by AI is a second opinion and always need to be verified by the doctor before starting the treatment and care. AI is not meant to replace human rather it should assist physician to give proper health care.

## Conflict of Interest

**None.**
